# High Trop-2 expression in pulmonary sarcomatoid carcinoma reveals antibody–drug conjugate targeting Trop-2 is a promising therapeutic approach

**DOI:** 10.1186/s12967-025-06888-3

**Published:** 2025-07-30

**Authors:** Xiaoying Qian, Chuanhong Luo, Chen Fang, Yongbin Wu, Weiwei Hong, Biao Yu, Guizhen Qin, Yan Yin, Xinyuan Yao, Xin Ye, Bingbiao Zhou, Chengsi Shu, Dengying Chen, Zhaoqing Li, Shanshan Wang, Yong Wang, Yong Li

**Affiliations:** 1 Department of Medical Oncology, The First Affiliated Hospital, Jiangxi Medical College, Nanchang University, 17 Yongwai Zheng Road, Nanchang, China; 2 Medical Innovation Center, The First Affiliated Hospital, Jiangxi Medical College, Nanchang University, 17 Yongwai Zheng Road, Nanchang, China; 3 Department of Internal Medicine, Dongxiang District People’s Hospital, 2 East Zhuangyuan Road, Fuzhou, China; 4 Department of Pathology, The First Affiliated Hospital, Jiangxi Medical College, Nanchang University, 17 Yongwai Zheng Road, Nanchang, China

**Keywords:** Pulmonary sarcomatoid carcinoma, Surgical, Trophoblast surface antigen 2, Intratumor heterogeneity, Antibody–drug conjugate

## Abstract

**Background:**

Several antibody–drug conjugates (ADC) targeting Trophoblast cell surface antigen 2 (Trop-2) have been developed, demonstrating significant therapeutic efficacy in triple-negative breast cancer and non-small cell lung cancer. However, the current expression of Trop-2 in pulmonary sarcomatoid carcinoma (PSC) and its clinical prognostic significance remains unclear.

**Materials and methods:**

Surgical tissue specimens diagnosed with PSC from January 2015 to May 2023 were retrospectively collected to detect Trop-2 expression using immunohistochemistry. The semi-quantitative H-score was employed to evaluate Trop-2 expression (< 10 is 0, 10–40 is 1 + , 41–140 is 2+, and 141–300 is 3+). We evaluated Trop-2 expression in PSC patients, comparing expression levels between the carcinomatous component (CaC) and the sarcomatous component (SaC), and analyzed their associations with clinicopathological characteristics and prognosis.

**Results:**

Thirty-five PSC patients receiving curative surgical resection were enrolled. The median disease-free survival (DFS) in PSC patients was 15.7 (95% CI 7.0–24.4) months, while the median overall survival was not reached. Positive expression of Trop-2 was observed in 31 (88.6%) PSC patients, the frequencies of 1+, 2+, and 3+ were 28.6%, 42.9%, and 17.1%, respectively. In 25 PSC patients with both CaC and SaC, we found a difference in Trop-2 expression between the two components (CaC vs. SaC, 100% vs. 56.0%). The intratumoral heterogeneity (ITH) of Trop-2 expression was not associated with clinicopathologic features. Patients in the CaC+/SaC+ group demonstrated significantly poorer DFS compared to the CaC+/SaC− group (12.5 months vs. > 60.0 months, *p* = 0.045). Multivariate Cox regression analysis indicated that an ECOG score of ≥ 1 (*p* = 0.004), stage II (*p* = 0.032), and CaC+/SaC+ (*p* = 0.030) were independently associated with a shorter DFS.

**Conclusion:**

The level of Trop-2 expression was high in PSC patients, and there is ITH in its expression. Targeting Trop-2 therapies may be a promising treatment for patients with PSC.

## Introduction

Pulmonary sarcomatoid carcinoma (PSC) is a rare and poorly differentiated subtype of non-small-cell lung cancer (NSCLC), accounts for less than 1% of NSCLC [1]. According to the latest 2021 World Health Organization (WHO) lung tumors classification, spindle cell carcinoma, and giant cell carcinoma are relegated to pleomorphic carcinoma [2]. Currently, PSC includes pleomorphic carcinoma (PLC), carcinosarcoma (CS), and pulmonary blastoma (PB). As PSC contains carcinomatous component (CaC) and the sarcomatous component (SaC), exhibits significant intratumoral heterogeneity (ITH), which leading to highly aggressive tumors and poor prognosis [3]. Without standard treatment, surgical resection remains the mainstay of curative treatment of PSC [4]. However, the prognosis of patients with PSC who undergo radical surgery is still worse than that of NSCLC after surgery [5–8]. Therefore, search for new effective therapeutic strategies is imperative to improve PSC patient’s prognosis.

Trophoblast cell surface antigen 2 (Trop-2) is a type I cell surface glycoprotein that belongs to the tumor-associated calcium signal transducer (TACSTD) family [9]. Elevated expression of Trop-2 was strongly associated with tumor metastatic, targeted, and immunotherapy resistance and poor survival outcomes in multiple malignancies [10–15]. Trop-2 is highly expressed in many tumor tissues, while its expression in normal tissues is limited [16]. In recent years, several antibody–drug conjugates (ADC) targeting Trop-2 have been developed, demonstrating significant therapeutic efficacy in triple-negative breast cancer (TNBC) and NSCLC [17–19]. These successes predict that ADC targeting Trop-2 has a broad application prospect in solid tumors. However, the current expression of Trop-2 in patients with PSC is still unknown.

While Trop-2 was highly expressed in the CaC, its expression in the SaC was significantly lower [17, 20, 21]. Nevertheless, whether intratumoral Trop-2 expression differs between CaC and SaC in PSCs remains unreported. Previous studies initially assessed the ITH in the CaC and SaC using Next-Generation Sequencing (NGS) [22]. However, due to the high price and technical complexity of NGS, analyses were limited to small sample sizes, precluding robust statistical correlations with clinical parameters. In contrast, immunohistochemistry (IHC) is cheaper and more accessible, enabling large-scale testing and facilitating the integration of Trop-2 expression data with clinical characteristics and prognosis outcomes. Notably, Trop-2 expression levels are predominantly evaluated via IHC in current research.

In this study, we employed IHC to detect Trop-2 expression in surgical specimens from patients with PSC and assessed its correlation with clinicopathological parameters and prognostic outcomes. Additionally, we analyzed Trop-2 expression heterogeneity between CaC and SaC within PSC and investigated its impact on postoperative survival. To our knowledge, this is the first study to report high expression of Trop-2 in PSCs and to clarify the presence of ITH. These findings provide novel insights into the ITH of PSCs and may offer a rationale for Trop-2-targeted therapeutic strategies.

## Materials and methods

### Patients and samples

PSC patients who underwent curative surgical resection from January 2015 to May 2023 at the First Affiliated Hospital of Nanchang University were enrolled. All tumor specimens were independently reviewed and confirmed by the experienced pathologist. PSC subtypes were classified according to the 2021 WHO classification, including PLC, CS, and PB. The patients with multiple primary malignancies were excluded. This study was approved by the Ethics Committee of the First Affiliated Hospital of Nanchang University.

### Data collection

All enrolled patients underwent curative surgical resection without prior neoadjuvant chemotherapy or radiotherapy. The postoperative pathological stage of PSC patients was determined based on the 9th edition of the TNM classification. The clinical information, including demographic characteristics, pathological features, tumor characteristics, and postoperative adjuvant therapy, was collected. Follow-up data were obtained through electronic medical records review and telephone interview. Disease-free survival (DFS) was defined as the time from surgery to the first tumor recurrence or death or deadline follow-up. Overall survival (OS) time was defined as the time from surgery to death or deadline follow-up. The deadline for the follow-up time of this study was March 31, 2024.

### IHC

The expression of Trop-2 in patients was detected by IHC. Briefly, 4um-thick formalin-fixed, paraffin-embedded tissue sections were dewaxed and rehydrated, followed by antigen retrieval with Tris/EDTA buffer (pH 9.0). Inhibition of endogenous peroxidase in specimens using 3% hydrogen peroxide. After incubation with Trop-2 primary antibody (1:2000, ab214488, Abcam) at 4 °C overnight, the slides were then incubated with secondary antibody at 37 °C for 20 min, followed by the DAB color reagent to develop the color reaction. Finally, slides were counterstained with hematoxylin and mounted for subsequent Trop-2 expression analysis.

### Evaluation of Trop-2 expression

Trop-2 expression was assessed independently by two experienced pathologists blinded to clinical outcomes using a semi-quantitative H-score. The H-score was calculated by multiplying the percentage of positive tumor cells and intensity of immunostaining [(1) no staining = 0; (2) mild = 1; (3) moderate = 2; and (4) strongly positive = 3)], which resulted in scores ranged from 0 to 300. A score of < 10 is 0, 10–40 is 1+, 41–140 is 2+, and 141–300 is 3+ [23]. Specimens were considered Trop-2 positive when the score ≥ 1+. Patients with PSC were scored for overall Trop-2 and further scored for CaC and SaC. The scoring results used for subsequent analysis.

### Statistical analysis

Intergroup comparisons of categorical variables were analyzed using Fisher’s exact test, and continuous variables were compared using the nonparametric test (Mann–Whitney U-test). The DFS and OS were performed using the Kaplan–Meier survival curve, and the log-rank test was used to assess differences in survival between groups. Multivariate survival analysis was performed using Cox regression model. All statistical analysis were performed using SPSS 26.0 software (SPSS, Chicago, IL). A double-sided *p* value < 0.05 indicated statistical significance.

## Results

### Clinical characteristics

Thirty-five patients with PSC who underwent curative surgical resection were enrolled in the present study. The detailed clinicopathological characteristics of PSC patients are presented in Table 1. The age of the patients ranged from 28 to 76 years, with a median age of 66 years. In all, 27 (77.1%) patients were male, 22 (62.9%) had a history of smoking, and 28 (80.0%) patients had an ECOG score of 0 at diagnosis. The majority of the tumors were located on the right side (22, 62.9%). Postoperative pathological results showed that 14 (40.0%) patients had tumors ≥ 5 cm and 7 (20.0%) had pleural invasion. Consistent with previous studies, PLC (30 cases, 85.7%) was the most common PSC subtype, and five further cases were CS (3, 8.6%) and PB (2, 5.7%). The proportions of postoperative pathological stage I, II, and III disease were 25.7, 31.4, and 42.9%, respectively. Fifteen out of 35 patients (42.9%) accepted postoperative adjuvant therapy, including chemotherapy, targeted therapy, or immunotherapy.

**Table 1:** Clinicopathological characteristics of patients with PSC who underwent curative surgical resection

Characteristics	No. of patients (%)
Median age (range)	66 (28–76)
Gender	
Male	27 (77.1)
Female	8 (22.9)
Smoking status	
Never	13 (37.1)
Former/current	22 (62.9)
ECOG at diagnosis	
0	28 (80.0)
≥ 1	7 (20.0)
Tumor size	
< 5 cm	21 (60.0)
≥ 5 cm	14 (40.0)
Primary location	
Left	13 (37.1)
Right	22 (62.9)
Histology	
PLC	30 (85.7)
CS	3 (8.6)
PB	2 (5.7)
Pathological stage	
I	9 (25.7)
II	11 (31.4)
III	15 (42.9)
Pleural invasion	
Yes	7 (20.0)
No	28 (80.0)
Postoperative adjuvant therapy	
Yes	15 (42.9)
No	20 (57.1)
Trop-2 expression	
0	4 (11.4)
1+	10 (28.6)
2+	15 (42.9)
3+	6 (17.1)

*PSC* Pulmonary sarcomatoid carcinoma, *ECOG* Eastern Cooperative Oncology Group, *PLC* pleomorphic carcinoma, *CS* carcinosarcoma, *PB* pulmonary blastoma, *Trop-2* Trophoblast cell surface antigen 2

### Postoperative survival analysis of patients with PSC

**Fig. 1:**
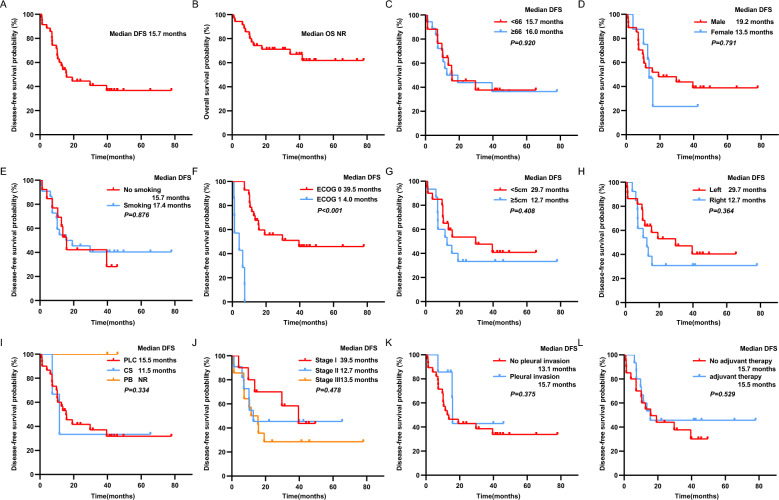
Postoperative PSC patients survival analysis. Kaplan–Meier analysis of DFS (**A**) and OS (**B**) in postoperative PSC patients. **C**–**L** The relationship between clinicopathological characteristics and postoperative DFS of patients with PSC. *PSC* pulmonary sarcomatoid carcinoma, *DFS* Disease-free survival, *OS* Overall survival, *NR* not reached, *ECOG* Eastern Cooperative Oncology Group, *PLC* pleomorphic carcinoma, *CS* carcinosarcoma, *PB* pulmonary blastoma

With a median follow-up duration of 31.3 months (1.0–77.9 months), 12 of the 35 PSC patients (34.3%) died at the final follow-up. The median DFS in PSC patients was 15.7 (95% CI 7.0–24.4) months (Fig. 1A), and the median OS was not reached (Fig. 1B). DFS in PSC patients was not associated with age, gender, or smoking status (*p* > 0.05) (Fig. 1C–E). Patients with an ECOG score of 0 had a significantly higher DFS than those with a score ≥ 1 (39.5 vs. 4.0 months), and the difference was statistically significant (*p* < 0.001) (Fig. 1F).

Patients with tumors located on the left side and those < 5 cm were associated with superior DFS over the right side and ≥ 5 cm, although there was no statistical difference (Fig. 1G, H). The DFS for PLC and CS were 15.5 and 11.5 months, respectively, and neither of the 2 patients with PB experienced recurrence or metastasis (Fig. 1I). Although there was no statistically significant difference in DFS by stage (*p* = 0.714), patients with stage I (39.5 months) had longer DFS than those with stage II (12.7 months) and III (15.5 months) (Fig. 1J). Pleural invasion and postoperative adjuvant therapy do not affect the patient's postoperative DFS (Fig. 1K, L).

### Correlation of Trop-2 expression with PSC patient clinicopathological characteristics and prognosis

**Fig. 2:**
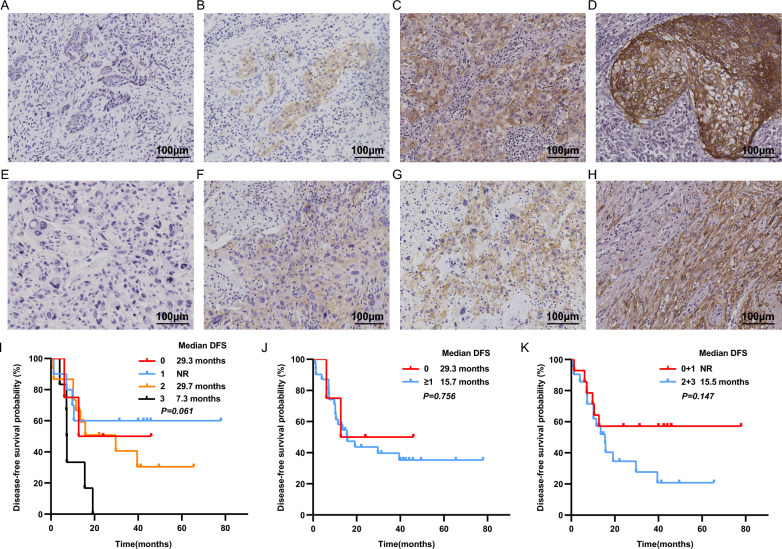
PSC patients with positive Trop-2 expression had shorter DFS. **A**–**D** Representative IHC images of CaC with Trop-2 staining intensity of 0 (**A**), 1 (**B**), 2 (**C**), and 3 (**D**). **E**–**H** Representative IHC images of SaC with Trop-2 staining intensity of 0 (**E**), 1 (**F**), 2 (**G**), and 3 (**H**). Scale bar: 100um; magnification at 200×. **I** Effect of different Trop-2 staining intensities on DFS in PSC patients. **J** DFS curves of PSC patients according to the Trop-2 positive expression (IHC score ≥ 1+). **K** DFS curves of PSC patients according to the Trop-2 high expression (IHC score ≥ 2+). Trop-2, Trophoblast cell surface antigen 2; PSC, pulmonary sarcomatoid carcinoma; DFS, Disease-free survival; NR, not reached; IHC, Immunohistochemistry.

The expression of Trop-2 was evaluated separately in the CaC (Fig. 2A–D) and SaC (Fig. 2E–H), and the overall expression levels were further analyzed. Trop-2 positive expression was observed in 31 (88.6%) patients with PSC (Table 1). Of the 4 (11.4%) patients without Trop-2 expression, 3 cases were consisted of complete spindle or giant cells, and 1 case was PB. The frequencies of Trop-2 expression as 1+, 2+ and 3+ were 28.6%, 42.9%, and 17.1%, respectively (Table 1). Notably, Trop-2 was positively expressed in all CaC, mostly 2+ to 3+ expression (24/25, 96.0%).

We further investigated the correlation between Trop-2 expression, clinicopathologic features, and DFS in PSC patients. Trop-2 positive expression was closely correlated with tumor size (Table 2). All the patients with negative Trop-2 expression had tumors with a size of ≥ 5 cm. The reason for this observation may be related to the more aggressive of the pure SaC or poorly differentiated tissue. However, no significant correlations were observed between Trop-2 expression and other clinicopathological features. Even though there was no statistical difference in DFS between different Trop-2 expression levels (*p* = 0.061) (Fig. 2I), patients with IHC 3+ (7.3 months) had worse DFS than those with IHC scores of 0, 1+, and 2+ (29.3, NR, and 29.7 months, respectively). The median DFS for patients without expression of Trop-2 was 29.3 months, while that of patients with positive Trop-2 expression was only 15.7 months (*p* = 0.756) (Fig. 2J). In addition, patients with IHC scores of 0 and 1+ patients had a longer DFS than with IHC scores of 2+ and 3+ (NR vs. 15.5 months *p* = 0.147) (Fig. 2K). Taken together, patients with no or low Trop-2 expression have a better prognosis.

**Table 2:** Correlation analysis between Trop-2 expression and clinicopathological characteristics in different PSC subgroups

Characteristics	All tumor site		*p* value	SaC		*p* value	CaC+/SaC		*p* value
	Negative N = 4	Positive N = 31		Negative N = 15	Positive N = 20		Negative N = 11	Positive N = 14	
Median age (range)	68 (65–72)	64 (28–76)	0.311	68 (55–76)	64 (28–73)	0.166	68 (55–76)	61.5 (28–72)	0.162
Gender			> 0.999			0.246			0.656
Male	3	24		10	17		7	11	
Female	1	7		5	3		4	3	
Smoking status			> 0.999			> 0.999			0.434
Never	1	12		6	7		5	4	
Former/current	3	19		9	13		6	10	
ECOG at diagnosis			> 0.999			0.672			0.604
0	3	25		13	15		10	11	
≥ 1	1	6		2	5		1	3	
Tumor size			0.019			> 0.999			0.233
< 5 cm	0	21		9	12		9	8	
≥ 5 cm	4	10		6	8		2	6	
Primary location			0.134			0.157			0.434
Left	3	10		8	5		5	4	
Right	1	21		7	15		6	10	
Histology			0.244			0.284			0.763
PLC	3	27		12	18		9	12	
CS	0	3		1	2		1	2	
PB	1	1		2	0		1	0	
Pathological stage			0.546			0.079			0.235
I	0	9		1	8		1	5	
II	2	9		7	4		5	3	
III	2	13		7	8		5	6	
Pleural invasion			> 0.999			0.112			0.623
Yes	1	6		5	2		3	2	
No	3	25		10	18		8	12	

*Trop-2* Trophoblast cell surface antigen 2, *PSC* Pulmonary sarcomatoid carcinoma, *ECOG* Eastern Cooperative Oncology Group, *PLC* pleomorphic carcinoma, *CS* carcinosarcoma, *PB* pulmonary blastoma, *SaC* sarcomatous component, *CaC* carcinomatous component

### Correlation of Trop-2 expression in SaC with clinicopathological characteristics and prognosis

In this study, the positive expression of Trop-2 in the SaC was only 57.1% (20/35, Table 2), and no cases exhibited an IHC score of 3+. To explore the association between Trop-2 expression in SaC and the clinicopathological characteristics and prognosis of patients. We divided the patients into SaC-negative and SaC-positive groups and analyzed the differences in clinicopathological features and prognosis between the two groups.

**Fig. 3:**
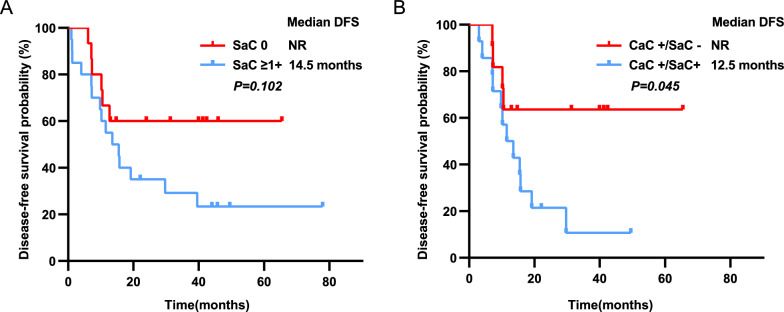
Intratumoral differences in Trop-2 expression are significantly associated with shorter DFS. **A** DFS curves of 35 PSC patients according to the Trop-2 positive expression in the SaC. **B** DFS curves of 25 PSC patients with both CaC and SaC according to the Trop-2 positive expression in SaC. *Trop-2* Trophoblast cell surface antigen 2, *DFS* Disease-free survival, *PSC* pulmonary sarcomatoid carcinoma, *SaC* sarcomatous component, *CaC* carcinomatous component, *NR* not reached

However, the differences in Trop-2 expression between the two groups were not associated with clinicopathological characteristics (Table 2). Although there was a obvious trend of worse DFS in those with positive Trop-2 expression (14.5 vs. > 60.0 months) in the SaC, DFS was not statistically significant between the two groups (*p* = 0.102) (Fig. 3A).

### Association of ITH of Trop-2 with clinicopathological characteristics and prognosis

We preliminary found that the positive expression of Trop-2 in the SaC of PSC patients was significantly lower compared to that in the CaC (57.1% vs. 100%). To further clarify the heterogeneity of Trop-2 expression between the CaC and SaC, we selected 25 patients who had both CaC and SaC for ITH analysis of Trop-2 expression. All of these 25 patients (100.0%) had Trop-2 positive expression in the CaC, while there were 56.0% patients had Trop-2 expression in the SaC (Table 2). Thus, we classified PSC patients into CaC+/SaC− and CaC+/SaC+ groups according to whether Trop-2 positive expression in the SaC.

The ITH of Trop-2 expression was not associated with clinicopathological characteristics (Table 2). It is worth noting that PSC patients in the CaC+/SaC+ group had a significantly worse prognosis compared to those in the CaC+/SaC− group (12.5 vs. > 60.0 months), and the difference was statistically significant (*p* = 0.045) (Fig. 3B). These findings demonstrate the existence of ITH in Trop-2 expression, and this ITH significantly affects the prognosis of patients with PSC.

### Multivariate analysis of DFS in PSC patients

Multivariate Cox regression analysis was employed to eliminate confounding factors that could potentially influence the DFS of PSC patients (Table 3). In the multivariate analysis involving 35 PSC patients, the results indicated that ECOG score of ≥ 1 (*p* < 0.001) and stage II (*p* = 0.019) were significantly associated with poor DFS. A further multivariate analysis was performed on 25 PSC patients who exhibiting ITH of Trop-2 expression. The results demonstrated that ECOG score of ≥ 1 (*p* = 0.004), stage II (*p* = 0.032), and CaC+/SaC+ (*p* = 0.030) were independently associated with a shorter DFS.

**Table 3:** Multivariate analysis of DFS in different PSC subgroups

Variable	N = 35		*p* value	N = 25		*p* value
	Category	HR(95% CI)		Category	HR(95% CI)	
Age	≥ 66 cm	0.994 (0.318–3.111)	0.992	≥ 66 cm	1.759 (0.320–9.662)	0.516
Gender	Female	1.581 (0.276–9.066)	0.608	Female	2.258 (0.216–23.616)	0.496
Smoking status	Former/current	4.386 (0.733–26.232)	0.105	Former/current	7.916 (0.655–95.632)	0.104
ECOG at diagnosis	≥ 1	153.715 (13.940–1695.033)	< 0.001	≥ 1	70.682 (4.035–1238.100)	0.004
Tumor size	≥ 5 cm	0.399 (0.041–3.876)	0.429	≥ 5 cm	0.215 (0.019–2.383)	0.210
Primary location	Right	0.661 (0.150–2.912)	0.584	Right	0.226 (0.037–1.390)	0.109
Histology	PLC	Ref		PLC	Ref	
	CS	0.208 (0.023–1.880)	0.162	CS	0.122 (0.009–1.582)	0.108
	PB	0.000	0.985	PB	0.000	0.988
Pathological stage	I	Ref		I	Ref	
	II	9.855 (1.462–66.404)	0.019	II	12.387 (1.248–122.997)	0.032
	III	3.437 (0.596–19.818)	0.167	III	6.772 (0.714–64.272)	0.096
Pleural invasion	No	1.197 (0.168–8.508)	0.857	No	2.993 (0.196–45.760)	0.431
Postoperative adjuvant therapy	No	3.511 (0.863–14.292)	0.080	No	1.092 (0.162–7.383)	0.928
Trop-2 expression	0	Ref		CaC+/SaC+	5.802 (1.185–28.399)	0.030
	1+	1.911 (0.102–35.680)	0.665			
	2+	2.869 (0.183–44.877)	0.453			
	3+	4.078 (0.326–51.023)	0.276			

*DFS* Disease-free survival, *PSC* Pulmonary sarcomatoid carcinoma, *ECOG* Eastern Cooperative Oncology Group, *Trop-2* Trophoblast cell surface antigen 2, *PLC* pleomorphic carcinoma, *CS* carcinosarcoma, *PB* pulmonary blastoma, *HR* hazard ratio, *Ref* reference, *SaC* sarcomatous component, *CaC* carcinomatous component

## Discussion

The impressive progress achieved with ADC therapies targeting Trop-2 altered the landscape for TNBC and NSCLC therapeutics [17–19]. This reinforces the notion that Trop-2 may be a new attractive therapeutic target for solid tumors. Hence, we investigated Trop-2 expression level and its relationship with clinicopathological characteristics and prognosis of surgical PSC patients. To the best of our knowledge, the present study is the first to report the high expression of Trop-2 in PSC and to confirmed the presence of ITH of Trop-2, with a significantly higher positivity rate in the CaC than in the SaC. Furthermore, our research found that positive expression of Trop-2 in the SaC was strongly associated with shorter DFS.

Targeted cancer therapies are capable of anticancer drugs targeting molecular targets on cancer cells and killing them precisely [16, 24]. These therapies require the targets to be present or highly expressed in tumor cells and less expressed in normal tissues to minimize the impact of the drug on normal tissues and to achieve a precise strike on the tumor. Trop-2 was initially identified on the surface of trophoblast cells [25]. In recent years, increasing studies have found that Trop-2 is highly expressed in multiple epithelial tumors, including lung, breast, gastric, and oral squamous cell carcinomas, while limited in normal tissues [16]. Currently, a variety of ADC targeting Trop-2 has been developed, including Sacituzumab govitecan and Datopotamab deruxtecan [17, 19].

A Phase I clinical trial of Datopotamab deruxtecan for the treatment of NSCLC included 180 patients who received a dosage of 4–8 mg/kg. These patients had previously undergone a median of three prior therapies. The trial reported an objective response rate of 26% and a median progression-free survival (PFS) of 6.9 months (NCT03401385) [19]. Almost all NSCLC in this clinical trial expressed Trop-2, which consistent with our findings that all CaC expressed Trop-2. Another clinical trial used sacituzumab govitecan to treat patients with advanced NSCLC who had also received a median of three prior therapies. Among the 47 patients with assessable responses, the ORR was 19%, andPFS was 5.2 months [17]. Nearly all (24/26, 92%) patients with advanced NSCLC in this clinical trial had Trop-2 expression of 2+ to 3+, which is similar to our results (CaC, 96%). The above studies demonstrated that ADC targeting Trop-2 still have promising antitumor efficacy even in heavily pretreated NSCLC patients. Regrettably, our study did not include PSC patients treated with Trop-2 ADC. As a result, we were unable to evaluate its therapeutic efficacy in this specific patient population. Furthermore, the limited number of NSCLC patients with weak or no Trop-2 staining precludes predicting the efficacy of this treatment and its correlation with clinicopathological characteristics based on Trop-2 expression levels.

In this study, we found that the differences in Trop-2 expression in PSC patients were only related to tumor size, and the tumors of patients without Trop-2 expression were all larger than 5cm. These findings may be related to the particular biological characteristics of the tumors. We speculate that the absence of Trop-2 expression can be ascribed to the deficiency or immaturity of epithelial components in purely sarcomatoid and fetus-like differentiation. Poorly differentiated tumors typically exhibit a more aggressive and have a larger tumor size.

In our study, PSC patients with high expression of Trop-2 had a shorter DFS compared to those with low or no expression. This is consistent with previous findings that patients with Trop-2 overexpression have a worse prognosis [10, 26]. Bessede et al. reported that NSCLC patients with high expression of Trop-2 had a poorer efficacy of immunotherapy than those with low expression (median PFS, 2.5 vs. 4.1 months, *p* < 0.001) [10]. In addition, patients with colorectal hepatic oligometastases with Trop-2 overexpression had poorer 3-year RFS (44.2% vs. 66.4%, *p* = 0.007) and 3-year OS (70.3% vs. 85.4%, *p* = 0.035) compared to patients with low expression [26].

There are limited studies of Trop-2 expression in sarcomas. A recent study reported the Trop-2 expression of sarcomatoid and rhabdoid bladder urothelial carcinoma to be 21.4% (6/28) and 14.3% (1/7), respectively [21]. In contrast, the overall Trop-2 positive expression of PSC patients in our study was 88.6%, significantly higher than the above studies. This difference may be due to different sites of tumorigenesis having different tumor biology. The above studies also illustrated a distinct gap in Trop-2 expression between the CaC and SaC.

Gu et al. found that Trop-2 is expressed in tumor tissues of osteosarcoma patients, and in vitro experiments clarified that Trop-2 promotes cell proliferation and migration in osteosarcoma through PI3K/AKT signaling [27]. This finding points out that the poor prognosis of osteosarcoma patients may be associated with Trop-2 expression. Furthermore, it has been reported that the prognosis of patients with PSC is worse than that of patients with NSCLC [4, 28–30]. Nevertheless, the prognostic impact of the Trop-2 expression of the SaC in PSC patients has not been reported. Therefore, in this study, we further analyzed the correlation of Trop-2 expression with clinicopathological characteristics and prognosis in the SaC of PSC patients. Our study preliminarily found that the positive expression of Trop-2 in the SaC was not associated with clinicopathologic characteristics but had shorter DFS. Therefore, we hypothesize that Trop-2 expression in the SaC is a risk factor for its poor prognosis and a potential therapeutic target. Additionally, we considered testing Trop-2 expression for sarcoma patients is necessary, which predicts that targeted therapy may be a potential treatment option for them. Further studies are required to explore the expression of Trop-2 and to evaluate the efficacy of targeted therapy in sarcomas.

Several studies have performed genetic testing on the CaC and SaC of PSC patients to elucidate their genomic origin and ITH [3, 22, 31]. These studies confirmed that the CaC and SaC shared numerous genomic alterations, indicating a common progenitor. EMT plays an important role in the SaC transformation process [3]. Thus, this study further analyzed the ITH of Trop-2 in 25 PSC patients with both the CaC and SaC. The results showed that Trop-2 was expressed in all CaC cases, while the positive rate of Trop-2 expression in the SaC was 56.0%, which confirmed the presence of ITH in Trop-2 expression. The positive rate of Trop-2 in the SaC of PSC patients was significantly higher than that of other sarcomas. We hypothesize that this phenomenon may be related to the common developmental origin of the CaC and SaC in PSC patients, and the SaC retains some of the characteristics of the CaC. In addition, a previous study reported that Trop-2 mRNA levels were reduced in a subpopulation of tumors exhibiting EMT characteristics, and a complete loss of Trop-2 protein expression was observed in the spindle cell component of sarcomatoid carcinomas [32]. It is plausible that the presence of EMT accounts for the lower expression of Trop-2 in the SaC compared to the CaC. Notably, our study demonstrated that the DFS of patients with Trop-2 expression in both CaC and SaC was significantly shorter than that of patients with CaC expression alone. This finding is consistent with the results of Liu et al. who argued that the CaC and SaC with a lower proportion of component-shared alterations have a longer DFS in PSC patients [22].

ADCs targeting Trop-2 utilize the expression of Trop-2 on tumor cell surfaces to achieve targeted killing of tumor cells. For patients with Trop-2 positive expression in both CaC and SaC, ADC therapies may be more effectively delivered to the tumor site, resulting in improved tumor killing ability. Given the ITH of Trop-2 expression in PSC patients, we speculate that the area without Trop-2 expression of the SaC may have low ADC drug delivery efficiency and suboptimal tumor killing effects. Shigehiro et al. reported that patritumab deruxtecan, an HER3-targeting antibody–drug conjugate, can effectively reache antigen-positive tumor sites and demonstrates similar drug concentrations in adjacent tumor tissues, facilitating the elimination of neighboring tumors through a bystander effect [33]. It remains uncertain whether Trop-2 ADC drugs can enhance drug delivery capacity and therapeutic efficacy through the bystander effect in PSC. Therefore, further research is essential to clarify this aspect.

ITH often leads to tumor resistance [34]. Chisato et al. discovered that ADCs with dual payloads can effectively treat refractory breast cancer, addressing tumor heterogeneity and drug resistance [35]. In melanoma, high expression of AXL contributes to resistance against MAPK pathway inhibitors [36]. The combined use of AXL ADC and BRAF/MEK inhibitors can tackle ITH, thereby enhancing treatment efficacy across different populations. Su et al. reported that alkaline phosphatase, placental-like 2 (ALPPL2) is a highly specific tumor cell surface antigen [37]. Moreover, they demonstrated the effectiveness of ADCs targeting ALPPL2 in killing tumor cells. This efficacy was observed in both in vitro and in vivo models of epithelioid and sarcomatoid mesothelioma. The studies mentioned above highlight the importance of using a combination strategy involving ADCs, and screening for antigens that are highly expressed in both the CaC and SaC tissues of PSC patients. This method is anticipated to enhance the delivery and therapeutic efficacy of ADC drugs while also minimizing drug resistance.

Although this study confirmed that Trop-2 expression was associated with poor prognosis in PSC patients, the majority of the results did not reach statistically significant. The small sample size was a notable limitation of this study. We preliminarily demonstrated the presence of intratumor heterogeneity in Trop-2 expression in postoperative specimens from PSC patients. Due to the small number of patients with postoperative adjuvant therapy, we cannot further investigate the impact of ITH on the efficacy of immunotherapy, targeted therapy, and other therapies, which is another limitation of this study. Hence, clinical studies with larger sample sizes are needed to validate the impact of Trop-2 expression in PSC patients with clinicopathologic characteristics and prognosis. Additionally, there is a lack of research on the mechanisms of Trop-2 overexpression and ITH in patients with PSC. A significant limitation of this study is its preliminary establishment of Trop-2 expression levels in PSC, as it lacks treatment data on Trop-2 ADC, preventing a clear understanding of treatment efficacy. We hope our study can provide a theoretical and practical basis for the subsequent research of Trop-2 ADC drugs in PSC treatment.

## Conclusion

Our study found that Trop-2 was highly expressed in PSC patients. Furthermore, these findings confirm the existence of heterogeneity in the Trop-2 expression between the CaC and SaC in PSC patients. Patients with Trop-2 expression in the SaC had significantly worse DFS. These results reveal that therapies targeting Trop-2 may be a promising treatment for patients with PSC. Given these findings, a prospective study to determine Trop-2 expression in PSC patients and explore the therapeutic efficacy of anti-Trop-2 ADC is warranted.

## Data Availability

The data from our hospital in the current study are available from the corresponding author on reasonable request.

## References

[1] Yendamuri S, Caty L, Pine M, et al. Outcomes of sarcomatoid carcinoma of the lung: a surveillance, epidemiology, and end results database analysis. Surgery. 2012;152(3):397–402. 22739072 10.1016/j.surg.2012.05.007

[2] Nicholson AG, Tsao MS, Beasley MB, et al. The 2021 WHO classification of lung tumors: impact of advances since 2015. J Thorac Oncol. 2022;17(3):362–87. 34808341 10.1016/j.jtho.2021.11.003

[3] Yang Z, Xu J, Li L, et al. Integrated molecular characterization reveals potential therapeutic strategies for pulmonary sarcomatoid carcinoma. Nat Commun. 2020;11(1):4878. 32985499 10.1038/s41467-020-18702-3 PMC7522294

[4] Robinson LA, Babacan NA, Tanvetyanon T, Henderson-Jackson E, Bui MM, Druta M. Results of treating primary pulmonary sarcomas and pulmonary carcinosarcomas. J Thorac Cardiovasc Surg. 2021;162(1):274–84. 32711968 10.1016/j.jtcvs.2020.03.179

[5] Zeng Q, Li J, Sun N, et al. Preoperative systemic immune-inflammation index predicts survival and recurrence in patients with resected primary pulmonary sarcomatoid carcinoma. Transl Lung Cancer Res. 2021;10(1):18–31. 33569290 10.21037/tlcr-20-960 PMC7867747

[6] Lococo F, Rapicetta C, Cardillo G, et al. Pathologic findings and long-term results after surgical treatment for pulmonary sarcomatoid tumors: a multicenter analysis. Ann Thorac Surg. 2017;103(4):1142–50. 28027731 10.1016/j.athoracsur.2016.08.114

[7] Felip E, Altorki N, Zhou C, et al. Adjuvant atezolizumab after adjuvant chemotherapy in resected stage IB-IIIA non-small-cell lung cancer (IMpower010): a randomised, multicentre, open-label, phase 3 trial. Lancet. 2021;398(10308):1344–57. 34555333 10.1016/S0140-6736(21)02098-5

[8] Herbst RS, Wu YL, John T, et al. Adjuvant osimertinib for resected EGFR-mutated stage IB-IIIA non-small-cell lung cancer: updated results from the phase III randomized ADAURA trial. J Clin Oncol. 2023;41(10):1830–40. 36720083 10.1200/JCO.22.02186 PMC10082285

[9] Qiu S, Zhang J, Wang Z, et al. Targeting Trop-2 in cancer: Recent research progress and clinical application. Biochim Biophys Acta Rev Cancer. 2023;1878(4): 188902. 37121444 10.1016/j.bbcan.2023.188902

[10] Bessede A, Peyraud F, Besse B, et al. TROP2 is associated with primary resistance to immune checkpoint inhibition in patients with advanced non-small cell lung cancer. Clin Cancer Res. 2024;30(4):779–85. 38048058 10.1158/1078-0432.CCR-23-2566 PMC10870116

[11] Tang W, Hu Y, Tu K, et al. Targeting Trop2 by Bruceine D suppresses breast cancer metastasis by blocking Trop2/β-catenin positive feedback loop. J Adv Res. 2024;58:193–210. 37271476 10.1016/j.jare.2023.05.012 PMC10982870

[12] Chen C, Chao Y, Zhang C, et al. TROP2 translation mediated by dual m(6)A/m(7)G RNA modifications promotes bladder cancer development. Cancer Lett. 2023;566: 216246. 37268280 10.1016/j.canlet.2023.216246

[13] Sun X, Jia L, Wang T, et al. Trop2 binding IGF2R induces gefitinib resistance in NSCLC by remodeling the tumor microenvironment. J Cancer. 2021;12(17):5310–9. 34335947 10.7150/jca.57711 PMC8317539

[14] Trerotola M, Jernigan DL, Liu Q, Siddiqui J, Fatatis A, Languino LR. Trop-2 promotes prostate cancer metastasis by modulating β(1) integrin functions. Cancer Res. 2013;73(10):3155–67. 23536555 10.1158/0008-5472.CAN-12-3266 PMC3655712

[15] Li Z, Jiang X, Zhang W. TROP2 overexpression promotes proliferation and invasion of lung adenocarcinoma cells. Biochem Biophys Res Commun. 2016;470(1):197–204. 26773504 10.1016/j.bbrc.2016.01.032

[16] Liu X, Deng J, Yuan Y, et al. Advances in Trop2-targeted therapy: Novel agents and opportunities beyond breast cancer. Pharmacol Ther. 2022;239: 108296. 36208791 10.1016/j.pharmthera.2022.108296

[17] Heist RS, Guarino MJ, Masters G, et al. Therapy of advanced non-small-cell lung cancer with an SN-38-anti-trop-2 drug conjugate, sacituzumab govitecan. J Clin Oncol. 2017;35(24):2790–7. 28548889 10.1200/JCO.2016.72.1894

[18] Rugo HS, Bardia A, Marmé F, et al. Overall survival with sacituzumab govitecan in hormone receptor-positive and human epidermal growth factor receptor 2-negative metastatic breast cancer (TROPiCS-02): a randomised, open-label, multicentre, phase 3 trial. Lancet. 2023;402(10411):1423–33. 37633306 10.1016/S0140-6736(23)01245-X

[19] Shimizu T, Sands J, Yoh K, et al. First-in-human, phase I dose-escalation and dose-expansion study of trophoblast cell-surface antigen 2-directed antibody-drug conjugate datopotamab deruxtecan in non-small-cell lung cancer: TROPION-PanTumor01. J Clin Oncol. 2023;41(29):4678–87. 37327461 10.1200/JCO.23.00059 PMC10564307

[20] Raji R, Guzzo F, Carrara L, et al. Uterine and ovarian carcinosarcomas overexpressing Trop-2 are sensitive to hRS7, a humanized anti-Trop-2 antibody. J Exp Clin Cancer Res. 2011;30(1):106. 22075385 10.1186/1756-9966-30-106 PMC3224774

[21] Brunelli M, Gobbo S, Malpeli G, et al. TROP-2, NECTIN-4 and predictive biomarkers in sarcomatoid and rhabdoid bladder urothelial carcinoma. Pathologica. 2024;116(1):55–61. 38482675 10.32074/1591-951X-937 PMC10938277

[22] Liu X, Wang F, Xu C, et al. Genomic origin and intratumor heterogeneity revealed by sequencing on carcinomatous and sarcomatous components of pulmonary sarcomatoid carcinoma. Oncogene. 2021;40(4):821–32. 33273725 10.1038/s41388-020-01573-9

[23] Liu H, Bai L, Huang L, et al. Bispecific antibody targeting TROP2xCD3 suppresses tumor growth of triple negative breast cancer. J Immunother Cancer. 2021;9(10): e003468. 34599021 10.1136/jitc-2021-003468 PMC8488747

[24] Tarantino P, Carmagnani Pestana R, Corti C, et al. Antibody-drug conjugates: Smart chemotherapy delivery across tumor histologies. CA Cancer J Clin. 2022;72(2):165–82. 34767258 10.3322/caac.21705

[25] Lombardi P, Filetti M, Falcone R, et al. Overview of Trop-2 in cancer: from pre-clinical studies to future directions in clinical settings. Cancers (Basel). 2023;15(6):1744. 36980630 10.3390/cancers15061744 PMC10046386

[26] Peng J, Ou Q, Deng Y, et al. TROP2 overexpression in colorectal liver oligometastases is associated with poor prognosis after liver resection. Ther Adv Med Oncol. 2019;11:1758835919897543. 35173815 10.1177/1758835919897543 PMC8842308

[27] Gu QZ, Nijiati A, Gao X, et al. TROP2 promotes cell proliferation and migration in osteosarcoma through PI3K/AKT signaling. Mol Med Rep. 2018;18(2):1782–8. 29845216 10.3892/mmr.2018.9083

[28] Gu L, Xu Y, Chen Z, Pan Y, Lu S. Clinical analysis of 95 cases of pulmonary sarcomatoid carcinoma. Biomed Pharmacother. 2015;76:134–40. 26653560 10.1016/j.biopha.2015.10.009

[29] Forde PM, Spicer J, Lu S, et al. Neoadjuvant nivolumab plus chemotherapy in resectable lung cancer. N Engl J Med. 2022;386(21):1973–85. 35403841 10.1056/NEJMoa2202170 PMC9844511

[30] Provencio M, Nadal E, González-Larriba JL, et al. Perioperative nivolumab and chemotherapy in stage III non-small-cell lung cancer. N Engl J Med. 2023;389(6):504–13. 37379158 10.1056/NEJMoa2215530

[31] Qin J, Chen B, Li C, Yan J, Lu H. Genetic heterogeneity and predictive biomarker for pulmonary sarcomatoid carcinomas. Cancer Genet. 2021;250–251:12–9. 10.1016/j.cancergen.2020.11.004 33217678

[32] Wang J, Zhang K, Grabowska D, et al. Loss of Trop2 promotes carcinogenesis and features of epithelial to mesenchymal transition in squamous cell carcinoma. Mol Cancer Res. 2011;9(12):1686–95. 21970857 10.1158/1541-7786.MCR-11-0241 PMC3243826

[33] Koganemaru S, Kawai T, Fuchigami H, et al. Quantitative analysis of drug distribution in heterogeneous tissues using dual-stacking capillary electrophoresis-mass spectrometry. Br J Pharmacol. 2023;180(6):762–74. 36377519 10.1111/bph.15988

[34] Dagogo-Jack I, Shaw AT. Tumour heterogeneity and resistance to cancer therapies. Nat Rev Clin Oncol. 2018;15(2):81–94. 29115304 10.1038/nrclinonc.2017.166

[35] Yamazaki CM, Yamaguchi A, Anami Y, et al. Antibody-drug conjugates with dual payloads for combating breast tumor heterogeneity and drug resistance. Nat Commun. 2021;12(1):3528. 34112795 10.1038/s41467-021-23793-7 PMC8192907

[36] Boshuizen J, Koopman LA, Krijgsman O, et al. Cooperative targeting of melanoma heterogeneity with an AXL antibody-drug conjugate and BRAF/MEK inhibitors. Nat Med. 2018;24(2):203–12. 29334371 10.1038/nm.4472

[37] Su Y, Zhang X, Bidlingmaier S, Behrens CR, Lee NK, Liu B. ALPPL2 is a highly specific and targetable tumor cell surface antigen. Cancer Res. 2020;80(20):4552–64. 32868383 10.1158/0008-5472.CAN-20-1418 PMC7572689

